# Lactate Dehydrogenase A is a potential prognostic marker in clear cell renal cell carcinoma

**DOI:** 10.1186/1476-4598-13-101

**Published:** 2014-05-05

**Authors:** Hala Girgis, Olena Masui, Nicole MA White, Andreas Scorilas, Fabio Rotondo, Annetta Seivwright, Manal Gabril, Emily R Filter, Andrew HA Girgis, Georg A Bjarnason, Michael AS Jewett, Andrew Evans, Sahar Al-Haddad, KW Michael Siu, George M Yousef

**Affiliations:** 1 The Keenan Research Center in the Li Ka Shing Knowledge Institute, St. Michael’s Hospital, Toronto M5B 1 W8, Canada; 2 Department of Laboratory Medicine, St. Michael’s Hospital, Toronto M5B 1 W8, Canada; 3 Department of Chemistry and Centre for Research in Mass Spectrometry, York University, 4700 Keele Street, Toronto M3J 1P3, Canada; 4 Department of Laboratory Medicine and Pathobiology, University of Toronto, Toronto M5S 1A8, Canada; 5 Department of Biochemistry and Molecular Biology, Faculty of Biology, University of Athens, 15701 Athens, Greece; 6 Department of Pathology, London Health Sciences Center and Western University, London N6A 5 W9, Canada; 7 Division of Medical Oncology and Hematology, Sunnybrook Odette Cancer Center, Toronto, ON M4N 3 M5, Canada; 8 Division of Urologic Oncology, Princess Margaret Hospital, University Health Network, Toronto, ON M5G 2 M9, Canada; 9 Department of Laboratory Medicine, University Health Network, Toronto, Canada

**Keywords:** Lactate dehydrogenase A, Prognosis, Renal cell carcinoma, Personalized medicine, Tumor markers, Proteomics, Pathology, Metastasis

## Abstract

**Background:**

Over 90% of cancer-related deaths in clear cell renal cell carcinoma (RCC) are caused by tumor relapse and metastasis. Thus, there is an urgent need for new molecular markers that can potentiate the efficacy of the current clinical-based models of prognosis assessment. The objective of this study is to evaluate the potential significance of lactate dehydrogenase A (LDHA), assessed by immunohistochemical staining, as a prognostic marker in clear cell renal cell carcinoma in relation to clinicopathological features and clinical outcome.

**Methods:**

We assessed the expression of LDHA at the protein level, by immunohistochemistry, and correlated its expression with multiple clinicopathological features including tumor size, clinical stage, histological grade, disease-free and overall survival in 385 patients with primary clear cell renal cell carcinoma. We also correlated the LDHA expression with overall survival, at mRNA level, in an independent data set of 170 clear cell renal cell carcinoma cases from The Cancer Genome Atlas databases. Cox proportional hazards models adjusted for the potential clinicopathological factors were used to test for associations between the LDHA expression and both disease-free survival and overall survival.

**Results:**

There is statistically significant positive correlation between LDHA level of expression and tumor size, clinical stage and histological grade. Moreover, LDHA expression shows significantly inverse correlation with both disease-free survival and overall survival in patients with clear cell renal cell carcinoma. Our results are validated by examining LDHA expression, at the mRNA level, in the independent data set of clear cell renal cell carcinoma cases from The Cancer Genome Atlas databases which also shows that higher lactate dehydrogenase A expression is associated with significantly shorter overall survival.

**Conclusion:**

Our results indicate that LDHA up-regulation can be a predictor of poor prognosis in clear cell renal cell carcinoma. Thus, it represents a potential prognostic biomarker that can boost the accuracy of other prognostic models in patients with clear cell renal cell carcinoma.

## Introduction

Renal cell carcinoma (RCC) is the most common type of malignant adult kidney tumors, accounting for more than 90% of all adult renal tumors. The incidence of renal cell carcinoma has been steadily rising in North America over the past decades [1]. About half of RCC patients are diagnosed with a localized tumor while 25% of patients present with locally invasive tumors, and the remaining 25% present with metastasis at the time of diagnosis, mainly to the bone and lung [2]. There are several histological sub-types of renal cell carcinoma which differ in incidence, biological behaviour and the potential metastasis. Approximately 75% of RCC patients have the clear cell renal cell carcinoma subtype (ccRCC) [3].

Over 90% of cancer associated mortality in RCC is due to metastasis, which is a complex process starting with local invasion, followed by intravasation, survival in the circulation, extravasation, initiation and maintenance of micro metastasis at distant sites, and finally, vascularisation of new tumor. The prognosis of RCC is quite variable. A number of prognostic models have been proposed [4], most of them are based only on clinical parameters but they all lack accuracy. In the new era of personalized medicine, better performing prognostic models are urgently required to better stratify patients for clinical trials and to provide more accurate clinical information that can significantly enhance decision making for patient management [5,6]. Improving the accuracy and discriminatory power of these models is likely to require identification and incorporation of multiple molecular markers [7]. A number of molecular markers for kidney cancer prognosis have been suggested but none of them have gained acceptance for clinical practice so far [8-11].

A number of genetic alterations and consequent metabolic changes are involved in metastatic progression of cancer [12]. The lactate dehydrogenase A (LDHA) protein, a target gene of c-Myc and hypoxia-inducible factor (HIF-1) located on the short p arm of chromosome 11 (11p15.4), is considered to be a critical branch point in metabolism of tumor cells [13-15]. Previous studies showed an association between up-regulation of LDHA and increased proliferation of tumor cells, particularly those with high malignant potential, and tumors that are poorly differentiated [16]. In oesophageal squamous cell carcinoma, knockdown of the expression of LDHA inhibited cell growth and cell migration in vitro as well as tumorigenesis in vivo [17]. It was also shown that knockdown of LDHA suppresses tumor growth and metastasis of human hepatocellular carcinoma [18]. On the other hand, inhibition of LDHA activity enhances mitochondrial respiration and decreases mitochondrial membrane potential which both compromises the ability of the tumor cells to proliferate under hypoxia and lead to apoptosis [19]. As the tumorigenicity of the LDHA-deficient cells was severely diminished, LDHA plays a key role in tumor maintenance [20].

Using quantitative proteomic analysis, we have recently identified a number of proteins, including LDHA, that are deregulated in metastatic compared to primary RCC [21], and showed that they are involved in pathways related to tumor progression and metastasis [22].

In the current study, we investigated the expression of LDHA in primary ccRCC and compared its expression with multiple clinicopathologic parameters including clinical stage, histological grade and tumor size. In addition, we examined the relationship between LDHA expression and patients’ survival. We show that high expression of LDHA can be considered as a predictor of poor prognosis in patients with ccRCC.

## Materials and methods

### Patient tissues and tissue micro array construction

Tissue microarrays (TMAs) were built using 385 tumor specimens from 10% buffered formalin fixed paraffin -embedded (FFPE) tissue blocks obtained with Research Ethics Board approval from the surgical pathology archives of St. Michael’s Hospital, University Health Network, and London Health Sciences Center between 2001–2009. All cases were primary clear cell renal cell carcinoma (ccRCC) and were reviewed by a pathologist. All new recognized entities including clear cell papillary, translocation carcinomas etc. were excluded from the analysis. Distribution of the numerical variables of the study population is shown in Table 1. Disease-free survival was defined as the time between the first surgical resection and disease recurrence. Overall survival was defined as the time between the first surgery for primary RCC and death for any reason. Diagnosis and selection of pure tumor areas were performed by a pathologist. Tumor grading was done according to the original Fuhrman grading system [23]. The two cores were obtained from two different blocks to account for tumor heterogeneity. Areas of necrosis were avoided. For 85 specimens, matched normal tissues from the same patient were available. Each specimen was represented by two 1 mm cores. Paraffin sections of the TMA were cut for immunohistochemistry (IHC) in 4 μm thickness.

**Table 1:** Distribution of numerical variables of the study population

			**Percentile**		
**Variable**	**Mean ± S.E.**^**a**^	**Range**	**25**^**th**^	**50**^**th **^**(Median)**	**75**^**th**^
Tumor size (cm)	5.9 ± 0.2	0.7 - 19.5	3.2	5.0	8.0
DFS (months)	50.0 ± 2.0	0.0 - 204.0	13.0	48.0	80.5
OS (months)	57.1 ± 1.9	0.0 - 216.0	24.00	61.0	86.0

^a^Standard error of the mean.

### Immunohistochemical staining and scoring

**Figure 1:**
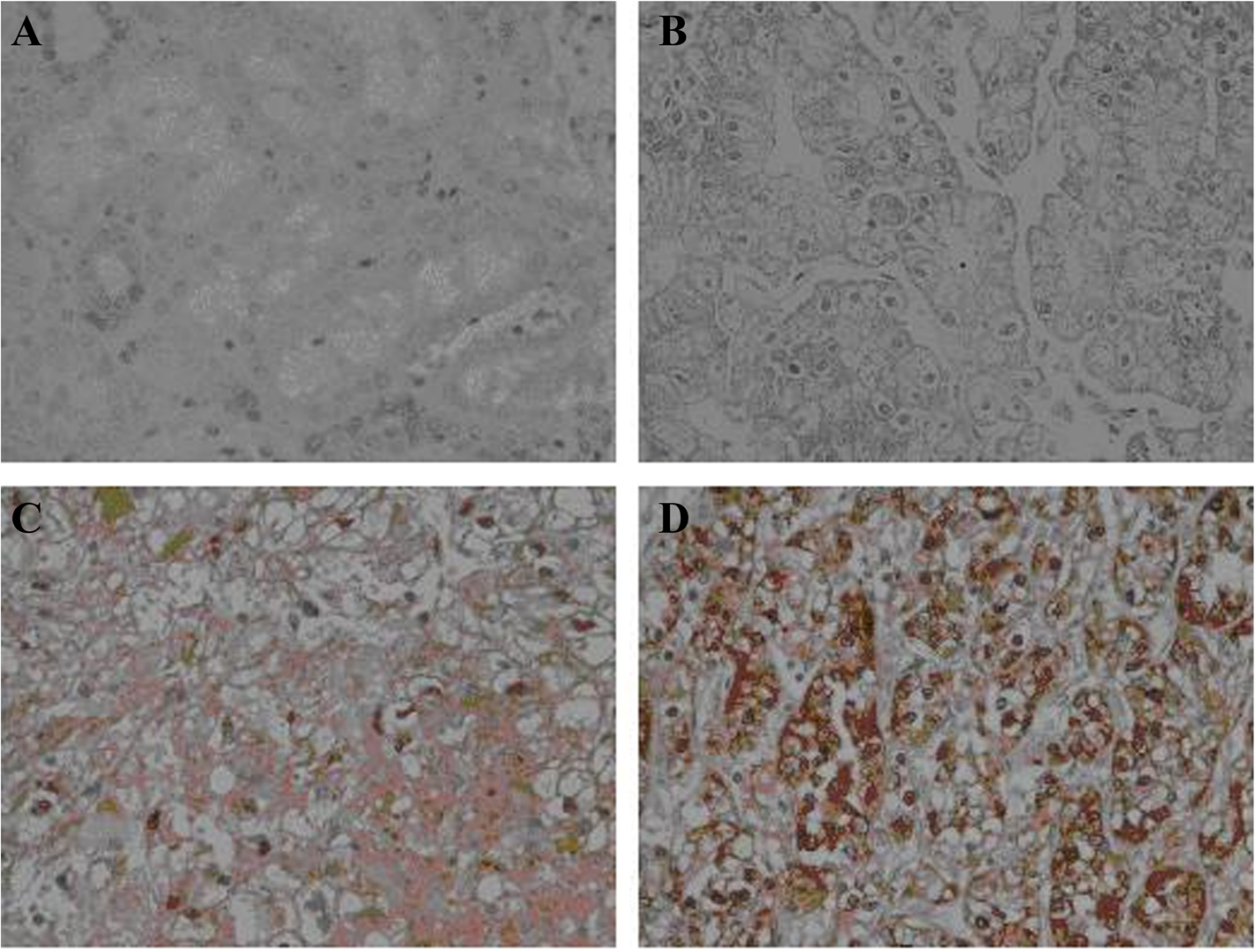
**Representative photomicrographs showing the expression of LDHA protein by immunohistochemistry. (A)** Normal kidney proximal tubular epithelium, **(B)** weak, **(C)** moderate, and **(D)** strong staining Clear Cell Renal Cell Carcinoma (all figures are original magnification X400).

Tumors were scored positive for LDHA if tumor cells showed definite nuclear and/or membranous staining and negative if tumor nuclei and cell membrane had no immunoreactivity. A combination of a proportion score and an intensity score was used to assess LDHA immunostaining: the proportion score (proportion of positive tumor cells on the studied section) was: 0: none, 1: 1-24%, 2: 25-49%, 3: 50–74, 4: ≥75%. The intensity score (intensity of staining by tumor cells) was: 0: none, 1: weak, 2: moderate, 3: strong. Three cases were used as calibrators to define intensity. A total score was obtained by the combining both scores. Cores were evaluated independently by two pathologists and controversial cases were assigned a consensus score after discussion. Representative micrographs of immunostaining are shown in Figure 1.

Two cores were evaluated for each patient, and the arithmetic average score was reported. Immunohistochemical staining was performed on paraffin-embedded specimen using a semi automated Discovery autostainer (Ventana Medical System, Inc., Tucson, AZ) and ChromoMapDAB multimer kit (Ventana). Tissues were exposed to the mouse monoclonal LDHA primary antibody (Abgent, Inc., Dan Diego, A; clone: 3H2; dilution 1:25) for 2 hours at room temperature, followed by 16- minutes using pre-diluted Ultra Map Rabbit horseradish peroxidase (Ventana). Antigen retrieval was performed using standard CC1. Replacement of the primary antibody with phosphate-buffered saline served as a negative control. In order to examine the effect of tumor heterogeneity on LDHA staining pattern, we compared its expression between the two cores of the same specimen. There was an 86% concordance in the expression levels of the two cores. Moreover, we compared expression between the TMA cores and whole slide staining in 20 cases. The degree of concordance was 90%.

### Statistical analysis

Fuhrman grading was assessed as that indicated in the pathology report of the case. Pathological staging was used for each patient. We performed Jonckheere-Terpstra Test in order to investigate the relationships between continuous variables and the three levels of the LDHA expression. Pearson Chi-square test or Fisher’s Exact Test were used to evaluate the associations between LDHA expression status and several clinicopathological variables. Univariate and Multivariate Cox proportional hazard regression analyses were used to estimate the prognostic significance of the LDHA in ccRCC patients. The Multivariate model was adjusted for patients’ sex, tumor size, histological grade and tumor stage. Cases with incomplete clinical information were excluded from related analysis. *P* values were calculated by the test for trend approach. Kaplan-Meier curves were also constructed in order to plot the percentage probability of patients’ DFS and OS, after their classification to LDHA-low, moderate or high expression.

### Bioinformatics analysis

Level 3 gene expression data (normalized gene expression data derived from the Cancer Genome Characterization Centre at the University of North Carolina using the Illumina HiSeq RNA Sequencing platform) for LDHA in ccRCC and normal kidney and overall survival data were obtained from The Cancer Genome Atlas (TCGA), available through the cBio Cancer Genomics Portal (http://www.cbioportal.org/public-portal). Tumors were selected with at least 80% tumor nuclei concentration and at most 5% necrosis generating a dataset of 170 cases. The X-tile algorithm was used to generate a prognostic optimal cut-off point to dichotomize LDHA mRNA expression as LDHA high expression and LDHA low expression using a Monte Carlo (*p* value <0.05). TCGA data types, platforms, and methods have been described previously. Twofold LDHA mRNA expression difference cut offs were generated for tumor versus geometric mean of normal kidney.

### mi RNA target prediction analysis

Target prediction analysis were performed using the target scan software (http://www.targetscan.org/).

### miRNA expression validation

To further validate our data we examined LDHA expression by qRT-PCR analysis using gene specific probes on 40 cases of primary ccRCC. PCR was performed according to the protocol suggested by the manufacturer.

## Results

### LDHA expression is up-regulated in primary ccRCC compared to normal kidney tissue

We compared the expression of LDHA protein between 85 matched normal/cancer specimens from the same patients. There was very weak/no expression of the LDHA protein in all normal proximal tubules of the kidney cortex (which is accepted in the literature as the structure of origin of ccRCC and is considered to be the appropriate normal control) [24]. ccRCC tissue from the same patients showed LDHA expression of variable frequency and intensity as described below. This is in keeping with recent literature and our previous data showing up-regulation of LDHA in RCC compared to normal tissue [25,26].

In order to validate our results, we evaluated LDHA expression, at the mRNA level; in an independent set of 170 cases of ccRCC (data are available from the TCGA database). In accordance with the protein expression, we observed increased LDHA mRNA levels in kidney cancer compared with normal renal cortex in 162 of 170 patients (95%). These results further confirm the potential prognostic utility of LHDA in this independent data set.

### The prognostic significance of LDHA protein in ccRCC

**Figure 2:**
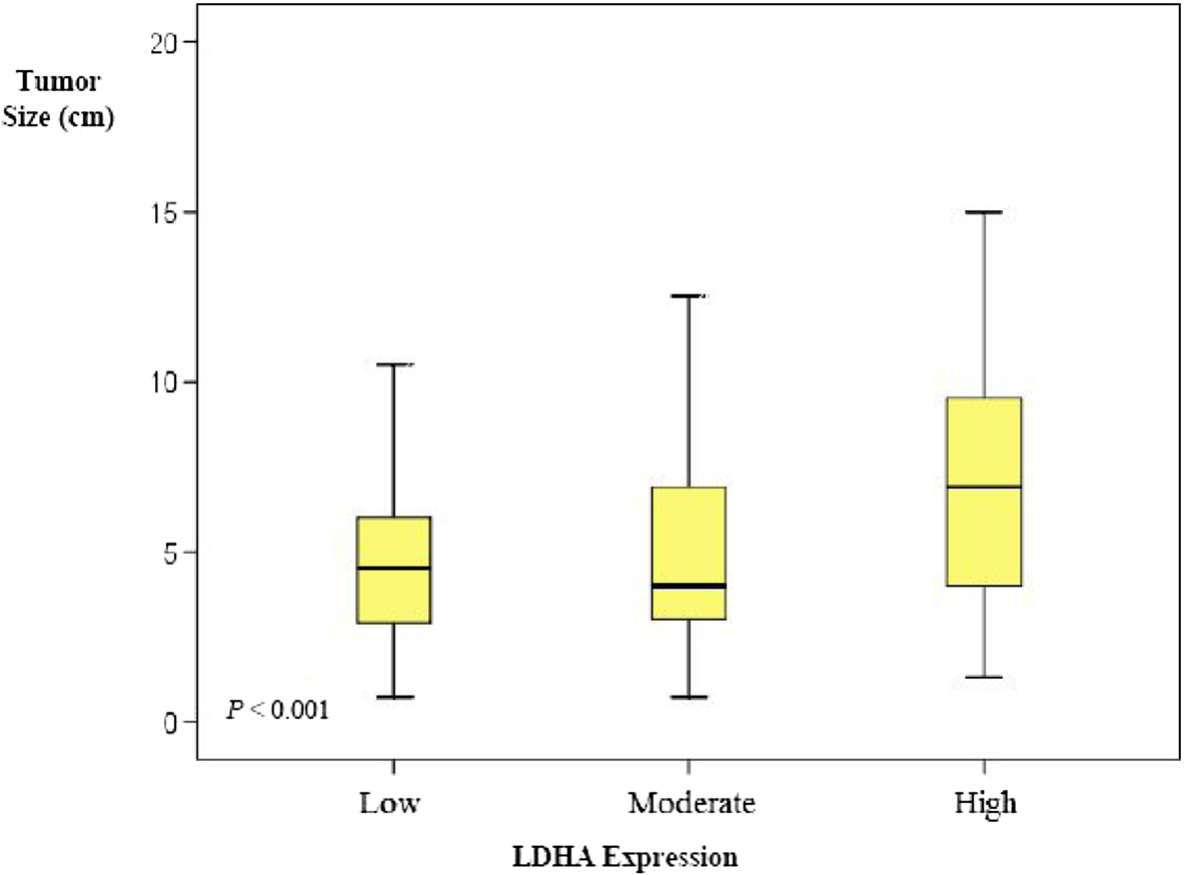
**Box plot representing LDHA expression levels in relation to tumor size.** There is a statistically significant association between the LDHA level of expression and tumor size; with larger tumors showing significantly higher proportion of LDHA expression (*p <* 0.001). Bold lines represent the median value (50th percentile) for each patient cohort. *P* value is calculated by “Jonckheere-Terpstra Test”.

The association between LDHA expression levels and other clinicopathological variables are summarized in (Table 2). There was a statistically significant association between the LDHA levels of expression and tumor size; with larger tumors showing significantly higher proportion of LDHA expression (*p <* 0.001) (Table 2 and Figure 2). There was also a stepwise increase in LDHA protein expression that is positively associated with tumor grade (*p <* 0.001) (Table 2). While 93% of grade I tumors expressed low to moderate levels of LDHA and LDHA high expression was only found in 6.5% of cases, in grade IV tumors 46% of cases showed low to moderate expression compared to 55% with high levels of expression. Moreover, there was a statistically significant stepwise increase in LDHA-high expression and tumor stage. While only 16% of stage I tumors showed high expression, the majority (79%) of stage IV tumors demonstrated LDHA-high expression (*p <* 0.001) (Table 2).

**Table 2:** Associations between LDHA expression levels and clinicopathological variables of patients

**Variable**	**Total**	**No. of patients (%)**		**LDHA-High expression**	***P*-value**^**a**^
		**LDHA-Low expression**	**LDHA-Moderate expression**		
**Tumor size**					
<5	196	35 (17.9%)	130 (66.3%)	31 (15.8%)	
≥5	149	23 (15.4%)	70 (47.0%)	56 (37.6%)	<0.001
x	40				
**Grade**					
I	31	5 (16.1%)	24 (77.4%)	2 (6.5%)	
II	180	29 (16.1%)	114 (63.3%)	37 (20.6%)	
III	103	18 (17.5%)	50 (48.5%)	35 (34.0%)	<0.001
IV	22	1 (4.5%)	9 (40.9%)	12 (54.5%)	
x	49				
**Stage**					
I	190	35 (18.4%)	124 (65.3%)	31 (16.3%)	<0.001
II	35	8 (22.9%)	18 (51.4%)	9 (25.7%)	
III	49	8 (16.3%)	27 (55.1%)	14 (28.6%)	
IV	33	0 (0%))	7 (21.2%)	26 (78.8%)	
x	78				
**Sex**					
Female	120	24 (20.0%)	75 (62.5%)	21 (17.5%)	0.051
Male	230	35 (15.2%)	128 (55.7%)	67 (29.1%)	
x	35				

Calculated using Pearson Chi-Square test.

x: Status is unknown.

The correlation of LDHA expression to the disease-free survival and overall survival were calculated using univariate and multivariate analyses (Table 3). Specimens were classified according to their LDHA level of expression as low, moderate, or high. Univariate analysis showed that patients who had tumors with high LDHA expression, exhibited significantly shorter disease-free survival than those who had tumors with low or moderate LDHA expression with a10 fold higher chances of relapse (hazard ratio (HR) = 10.23; *p* < 0.001). Also, LDHA higher expression was associated with significant shorter overall survival with 4 times chances of death. (HR = 4.04; *p* = 0.026). In the multivariate analysis, LDHA continued to show significant correlation between high expression and shorter disease-free survival after controlling for other variables (HR = 4.3; *p* = 0.007). There was also a trend of positive association between high LDHA expression and poor overall survival, although this did not reach statistical significance after controlling for other variables (HR = 3.46*, p* = 0.1).

**Table 3:** Univariate and multivariate analyses of LDHA expression and patients’ survival

**Variable**	**Disease-free survival**			**Overall survival**		
	**HR**^**a**^	**95% CI**^**b**^	***P*-value**	**HR**^**a**^	**95% CI**^**b**^	***P*-value**
**Univariate analysis (N = 338)**						
**LDHA expression**						
Low	1.00			1.00		
Moderate	1.12	0.37-3.39	0.83	0.97	0.26-3.59	0.96
High	10.23	3.68 - 28.41	<0.001	4.04	1.18 -13.85	0.026
**Stage (nominal)**	7.10	4.29 - 11.75	<0.001	3.57	1.75 - 7.31	<0.001
**Histological grade(nominal)**	4.27	2.65 - 6.88	<0.001	3.78	1.92 - 7.44	<0.001
**Multivariate analysis**^**c **^**(N = 279)**						
**LDHA expression**						
Low	1.00			1.00		
Moderate	0.90	0.29 - 2.78	0.85	0.92	0.19 - 4.51	0.92
High	4.30	1.48 - 12.53	0.007	3.46	0.73 - 16.36	0.12
**Tumor size (nominal)**	1.70	0.86 - 3.37	0.13	0.69	0.26 - 1.87	0.46
**Stage (nominal)**	3.35	1.89 - 5.94	<0.001	1.86	0.78 - 4.41	0.16
**Histological grade(nominal)**	2.11	1.13 - 3.96	0.02	3.17	1.17 – 8.60	0.02
**Sex**	0.70	0.40 - 1.24	0.23	1.14	0.44 - 2.95	0.78

^a^Hazard ratio, estimated from Cox proportional hazard regression model.

^b^Confidence interval of the estimated HR.

^c^Multivariate models were adjusted for tumor size, histological grade, sex and tumor stage.

**Figure 3:**
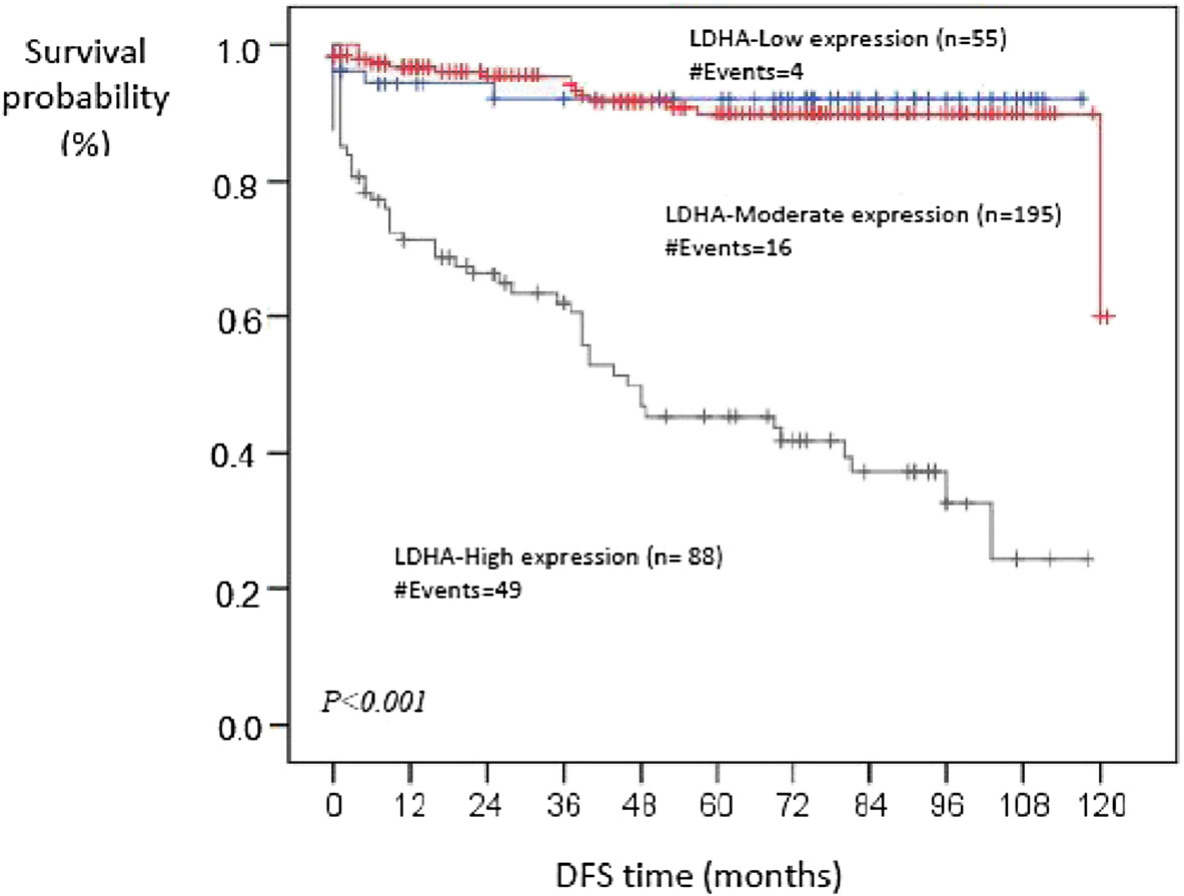
**Kaplan-Meier curve for DFS of patients with low, moderate and high expression of LDHA protein.** Patients in the high LDHA expression arm have a statistically significant decrease in disease-free survival *(p* = 0.001) compared to those in the low LDHA expression arm.

**Figure 4:**
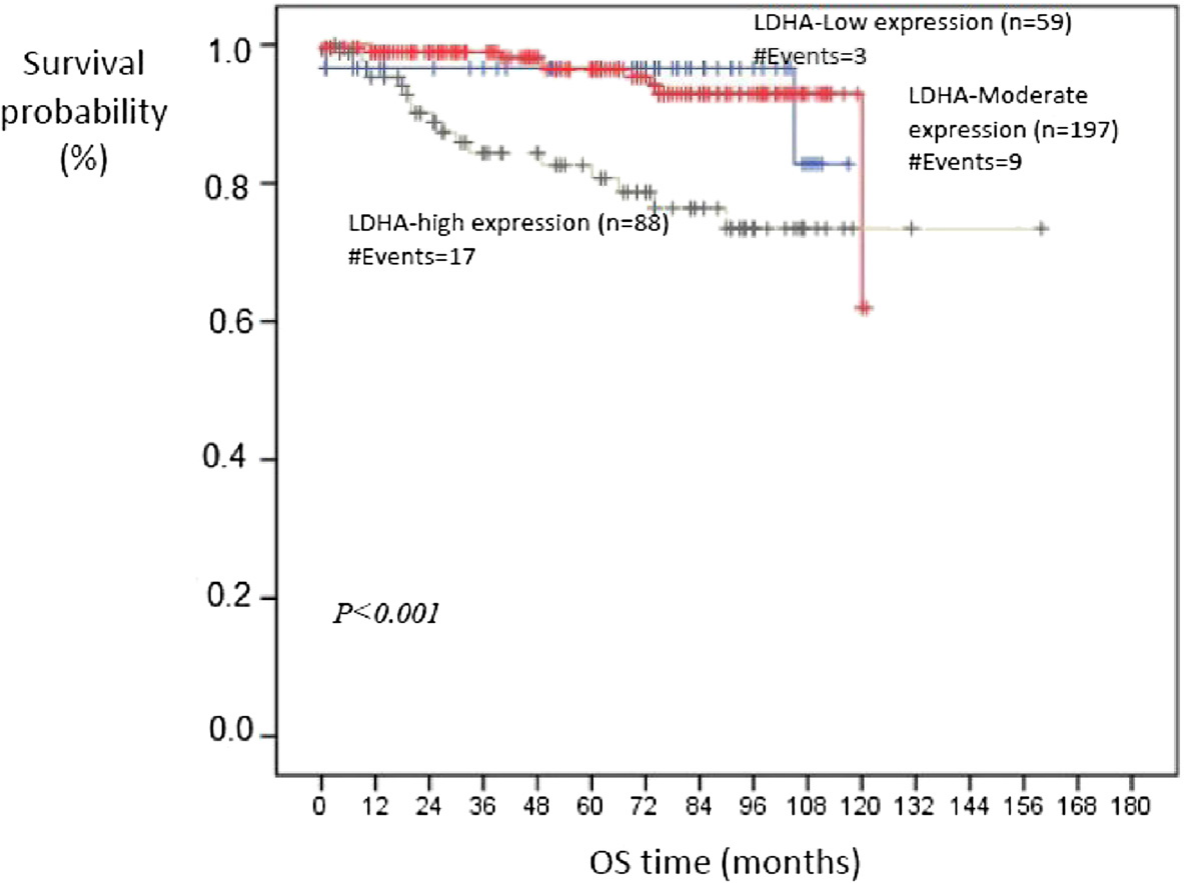
**Kaplan-Meier curve for OS of patients with low, moderate and high expression of LDHA protein.** Patients in the high LDHA arm have a statistically significant decrease in overall survival (*p* < 0.001) compared to those in the low and moderate LDHA expression arm.

Kaplan-Meier survival curves indicated that patients in the high LDHA expression arm have a statistically significant decrease in disease-free survival *(p* = 0.001) compared to those in the low LDHA expression arm (Figure 3). Also, high LDHA expression was associated with significant decrease in overall survival compared to low or moderate expression (*p* < 0.001) (Figure 4). Taken together, these data show that LDHA expression can be used as a strong independent predictor of poor prognosis in patients with ccRCC.

### Prognostic value of LDHA at the mRNA level

**Figure 5:**
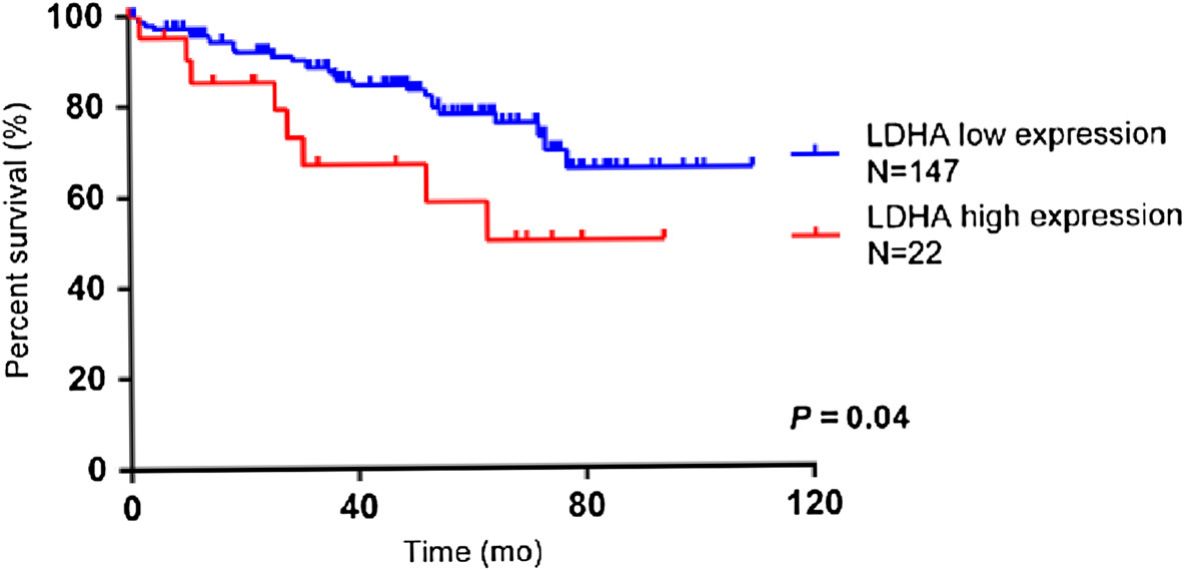
**Kaplan-Meier survival curve for overall survival of patients with low and high expression of LDHA.** mRNA data was obtained from the Cancer Genome Atlas (TCGA) dataset. On the basis of LDHA expression, patients are classified as LDHA high expression and LDHA low expression. Similar to the results of imunohistochemical analysis, the curve indicates a statistically significant reduction in overall survival with higher LDHA mRNA expression (*p* = 0.04).

We also examined the prognostic significance of LDHA expression in ccRCC at the mRNA level using the TCGA dataset. On the basis of LDHA expression, patients were classified as LDHA high expression and LDHA low expression using an optimized cut-off value of the 87^th^ percentile (see Materials and Methods), and the resulting groups included 22 LDHA high expression patients and 147 low expression patients. Similar to the results of IHC analysis, we detected a statistically significant reduction in overall survival with higher LDHA mRNA expression (*p* = 0.04) (Figure 5).

### LDHA mRNA expression validation

We further validated our results using another independent set of 40 cases of primary ccRCC at mRNA level. Patients with higher LDHA expression were associated with a significantly worse prognosis compared to those who are LDHA negative (*p* = 0.023). (Data are not shown).

### Mechanisms of LDHA up-regulation in ccRCC

To explore potential mechanisms of LDHA activation, we have recently conducted genome-wide copy number profiling of ccRCC specimens and have observed whole chr11 gain in 5% of specimens [27]. To validate our results, we quarried LDHA copy number gain on the ccRCC data set from TCGA. A total of 170 ccRCCs were analyzed (see Materials and Methods). LDHA copy number gain was observed in 9 of 170 specimens (5%). These findings indicate that copy number gain could be responsible, at least in part, for LDHA up-regulation in ccRCC.

Also, in order to explore other mechanisms that can results in the differential expression of LDHA in renal cell carcinoma, we performed target prediction analysis using TargetScanHuman 6.2 (http://www.targetscan.org/). Our analysis showed that LDHA is a predicted target of miR-449a, miR-449b, miR-34a and miR-34c-5p.

We next compared the expression of LDHA and its targeting miRNAs between RCC and normal kidney tissues. We observed an inverse correlation between LHDA and miR-449a and miR-449b, where the levels of these two miRNAs were down-regulated in primary ccRCC compared to normal kidney [28,29] while LDHA was up-regulated. Taken together, these data point indicate that these miRNAs can be responsible, at least in part, for the LDHA up-regulation in ccRCC.

In order to further explore potential mechanisms that can be responsible for LHDA up-regulation in aggressive RCC correlated its expression between primary and metastatic RCC. We identified an inverse correlation between miR-34a and LDHA between primary and metastatic ccRCC, indicating that miR34a down-regulation in metastatic disease [28] can release its inhibitory effect on LDHA with subsequent up-regulation.

## Discussion

Clear cell Renal Cell Carcinoma is the most common subtype of renal cell carcinoma with increasing incidence, high metastatic potential and high mortality rate. It would be useful to understand the process of metastasis in ccRCC and to discover novel parameters that impact the risk of recurrence and response to specific therapies.

Earlier studies showed that cancer cells preferentially use glycolysis to produce adenosine triphosphate, even in the presence of normal levels of oxygen. The abnormalities that occur in energy metabolism of cells are a fundamental aspect of cancer (Warburg effect phenomenon) [30-32]. It has been established that in Fumarate Hydratase (FH)-deficient kidney cancer, there is impaired oxidative phosphorylation with metabolic shift to aerobic glycolysis to generate ATP required for the increased energy demand of the rapidly proliferating tumor cells [33]. This process requires the uptake of glucose and the generation of lactate through LDHA. Increased lactate production eventually leads to glutamine dependent reductive carboxylation, rather than oxidative metabolism. Metabolic changes have also been reported in RCC [22,34]. Glutamine is the major source for the increased fatty acid synthesis required for rapid proliferation of cancer cells. LDHA plays a key role in aerobic glycolysis as it is involved in the metabolism of the two major nutrients glucose and glutamine. Accordingly, LDHA is critical in maintenance and progression of tumors. Previous studies confirmed the significant higher expression of LDHA in RCC compared to the corresponding normal kidney tissue [35,36]. On the other hand, it was also proven that LDHA inhibition results in increasing apoptosis of the tumor cells in FH-deficient kidney cancer and is considered in therapeutic strategy for patients with kidney cancer [37-39].

The correlation between LDHA expression and the tumor grade should be interpreted with caution because the current system of kidney cancer grading relies on assigning the grade of the tumor according to the area of the highest grading not according to the grading of the majority of the tumor.

Our results demonstrate the potential prognostic value of LDHA in ccRCC by detecting its significantly higher expression in patients with advanced clinical stage and histological grade. We also found that LDHA level of expression in primary RCC is directly proportional with the tumor size and is associated with significant decrease of both disease free survival and overall survival. We validated our results at the mRNA level using an independent data set from TCGA databases. The results confirm the prognostic utility of LDHA in ccRCC. Our results are in agreement with previous clinical findings. A commonly used model to predict risk in metastatic RCC, defined by Motzerat Memorial Sloan-Kettering, includes high serum LDH as one of the prognostic factors [40]. This model has been validated [41]. Another recent study showed that LDH and performance status outperformed several other variables that are part of prognostic models in patients with brain metastases [42].

We have also shown that copy number aberration was only observed in 5% of ccRCC cases. This is consistent with the recent data published by our group and others [7,27,43,44]. This was validated by our recent study and others.

At present, there are few proposed molecular prognostic markers described in kidney cancer [45]. They include CD31, EDNRB and TSPAN7 [46], carbonic anhydrase 9 [47], factor inhibiting hypoxia- inducible factor (FIH) [48], VHL protein alterations [49], miR-21 expression [8], microvascular density [50], and the chromatin remodelling gene ARID1A [51]. Integration of LDHA appears to be a useful additional way to identify poor prognosis in kidney cancer patients.

In conclusion, we provide strong evidence for the potential utility of LDHA as a prognostic marker for both disease free and overall survival in RCC. Our data was validated in two independent sets, both in the mRNA and protein levels of expression. Integration of LDHA expression levels in multiparametric model might enhance its performance. In addition to be a prognostic marker, our data paves the road for investigating LDHA as a potential therapeutic target in RCC.

## Abbreviations

LDHA: Lactate dehydrogenase A; ccRCC: Clear cell renal cell carcinoma.

## Competing interests

The authors declare that they have no competing interests.

## Authors’ contributions

HG interpreted the IHC results, OM, NMAW, FR performed the immunohistochemistry, AS performed the statistical analysis, ALS, MG, ERF, AE, SA constructed the tissue microarray and obtained the clinical parameters and survival information, AHAG performed the in silico analysis, GAB, MASJ, KWMS, and GMY conceived of the study, and participated in its design and coordination. All authors contributed to manuscript writing and read and approved the final manuscript.
